# Abscisic Acid as an Emerging Modulator of the Responses of Plants to Low Oxygen Conditions

**DOI:** 10.3389/fpls.2021.661789

**Published:** 2021-04-26

**Authors:** Miguel González-Guzmán, Aurelio Gómez-Cadenas, Vicent Arbona

**Affiliations:** Laboratori d’Ecofisiologia i Biotecnologia, Departament de Ciències Agràries i del Medi Natural, Universitat Jaume I, Castelló de la Plana, Spain

**Keywords:** abscisic acid, anoxia, ethylene, gibberellins, hypoxia, nitric oxide, soil flooding

## Abstract

Different environmental and developmental cues involve low oxygen conditions, particularly those associated to abiotic stress conditions. It is widely accepted that plant responses to low oxygen conditions are mainly regulated by ethylene (ET). However, interaction with other hormonal signaling pathways as gibberellins (GAs), auxin (IAA), or nitric oxide (NO) has been well-documented. In this network of interactions, abscisic acid (ABA) has always been present and regarded to as a negative regulator of the development of morphological adaptations to soil flooding: hyponastic growth, adventitious root emergence, or formation of secondary aerenchyma in different plant species. However, recent evidence points toward a positive role of this plant hormone on the modulation of plant responses to hypoxia and, more importantly, on the ability to recover during the post-hypoxic period. In this work, the involvement of ABA as an emerging regulator of plant responses to low oxygen conditions alone or in interaction with other hormones is reviewed and discussed.

## Introduction

**Figure 1:**
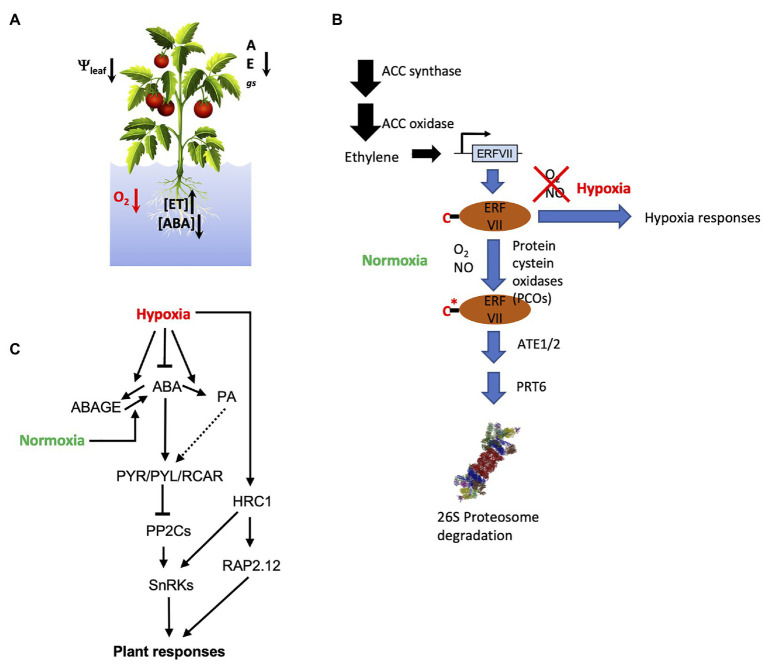
Plant responses to oxygen deprivation: **(A)** Physiological and hormonal responses to soil flooding where gs is stomatal conductance, E is transpiration rate, and A is the photosynthesis rate; **(B)** ethylene and N-degron pathway coordinate the tissue response to both normoxia and hypoxia O_2_ conditions, C^*^ indicates the modification of Cys to Cys-sulfinic acid in the presence of O_2_; and **(C)** molecular players involved in the interaction of the *core* abscisic acid (ABA) signaling with O_2_ conditions at molecular level in higher plants.

The alteration of rainfall regimes caused by climate change will generate extremely arid world regions and others with increased pluviometry that will waterlog populated areas and arable lands. Waterlogging is a stress condition that restricts access of plant tissues to CO_2_ and O_2_ incurring in hypoxic or anoxic conditions, depending on the degree of oxygen depletion. In aerated organs of waterlogged plants, hypoxic conditions induce a reduction of gas exchange parameters and a progressive inhibition of photosynthesis rate (Figure 1A) together with an inhibition of the mitochondrial electron transport chain leading to a reduction in ATP production and a subsequent elevation of reactive oxygen species (ROS) production. Prolonged submergence periods followed by reoxygenation, the post-anoxic period, enhance some of the adverse effects of anoxia with devastating results (Blokhina et al., 2003). However, some plant species have developed adaptative traits to flooding stress allowing them to occupy flooded habitats. Several plant species from the genera *Oryza*, *Rumex*, and *Nasturtium* have developed flooding survival strategies, which are not normally present in cultivated species, but could be introgressed in crops to maintain yield. For instance, some survival flooding strategies of wild *Oryza* species such as the formation of aerenchyma are already present in several cultivated rice varieties. Moreover, as floodwaters recede, plants undergo a reoxygenation period to reach the normal cellular oxygen conditions with a great impact in the physiology of the plant which had already adapted to low-O_2_ conditions. Sudden re-exposure to normal oxygen conditions and subsequent re-activation of aerobic metabolism causes extensive plant damage attributed to excessive ROS formation. Moreover, de-submergence reveals the limitation on water absorption associated to reduced root hydraulic conductance due to waterlogging which leads to water stress on aerial plant tissues. Hence, the responses of plants to low-O_2_ and subsequent reoxygenation involve several processes that must be also considered.

## Oxygen As a Gaseous Signal

Oxygen shortage to plant tissues is usually associated to total or partial submergence due to (i) normal agricultural practices (e.g., in rice cultivation, flooding of lands to prevent outgrowth of competitor weeds) or (ii) heavy rains that flood flatlands and poorly drained soils. Molecular oxygen, O_2_, is essential to all aerobic life as the ultimate electron acceptor in the mitochondrial electron transport chain (ETC) which availability modulates electron flow through ETC and ATP biosynthesis (Bailey-Serres and Voesenek, 2008). Moreover, O_2_ participates in different essential plant biochemical pathways (Holdsworth and Gibbs, 2020). Therefore, to ensure a convenient response to environmental oxygen levels, protein oxygen sensors exist in plants and animals known as hypoxia-responsive transcription factors which stability is directly linked to O_2_ levels: in the presence of oxygen, these transcription factors are ubiquitinated and rapidly degraded by 26S proteasome. In angiosperms, this role is partially overtaken by group VII of ETHYLENE RESPONSE FACTORS (ERFVIIs) that control anaerobic gene expression under hypoxia (Holdsworth and Gibbs, 2020). This group of transcription factors, contain specific N-terminal residues which, following co-translational Met excision, are subsequently modified by PLANT CYSTEINE OXIDASES (PCOs) that convert N-terminal Cys to Cys-sulfinic acid in the presence of O_2_, subsequent arginylation by ARGININE TRANSFERASE (ATE) provides an adequate target for the E3 ligase PROTEOLYSIS 6 (PRT6). This pathway is known as the N-end rule or N-degron pathway (Gibbs et al., 2011; Holdsworth and Gibbs, 2020), which, in higher plants, requires not only O_2_ but also nitric oxide (NO) and ethylene (ET; Gibbs et al., 2014; Figure 1B).

In *Arabidopsis thaliana*, five ERFVIIs have been identified: *RAP2.2*, *RAP2.3*, *RAP2.6*, *RAP2.12*, *HRE1*, and *HRE2*, which control hypoxia survival responses mediated by the increase of pyruvate decarboxylase (PDC), alcohol dehydrogenase (ADH), and sucrose synthase (SuSY) activities, enabling switch from aerobic to anaerobic metabolism (Gibbs et al., 2011). ET induces ERFVIIs gene expression under hypoxia but their stability and, hence, function at low-O_2_ levels are defined by the N-degron pathway (Figure 1B). Besides hypoxia stress responses, this pathway also regulates tissue-dependent and developmental functions in the so-called hypoxic niches by targeting different proteins (Labandera et al., 2021), constituting plastic developmental cues that regulate key physiological processes such as germination, photomorphogenesis, or plant immune responses (Gibbs et al., 2014; Abbas et al., 2015; Labandera et al., 2021). In addition, five different PCOs activities with specific gene expression profiles and substrate specificities have been described in Arabidopsis, adding more complexity to the O_2_ sensing activity of the N-degron pathway constituting a plausible interaction hub between different signaling pathways (White et al., 2018; Masson et al., 2019). Other protein activities such as prolyl hydroxylases, thiol oxidases, or some lysine demethylases have been suggested as putative O_2_ sensors (Strowitzki et al., 2019; Holdsworth and Gibbs, 2020). Moreover, it is worth to mention the identification of a gene coding for a Raf-like MAPKKK named *HYDRAULIC CONDUCTIVITY OF ROOT 1* (*HCR1*) which integrates the reduction of the root hydraulic conductivity coded by the K^+^ availability and the root O_2_ status. This suggests the existence of ion channels in the plasma membrane acting as putative O_2_ sensors along with other elements involved in decoding and integrating these signals (Shahzad et al., 2016). In addition, the acquisition of low-O_2_ tolerance could be associated to hypoxia-dependent chromatin modifications resulting from exposure of plants to consecutive hypoxic events that could be transmitted to the progeny. To this respect, the reduction of histone demethylase KDMA5A and 6A activities under hypoxic conditions increase the histone methylation status and regulate the hypoxia-dependent gene expression (Batie et al., 2019; Chakraborty et al., 2019).

## Physiological Responses of Plants To Soil Flooding and Recovery Conditions

In spite of the fact that plant adaptation to flooding conditions is a species-dependent process, two different strategies can be distinguished: (i) low-O_2_ quiescent syndrome (LOQS) characterized by the suppression of growth and (ii) low-O_2_ escape strategy (LOES) involving growth promotion. Plants that adopt the LOQS strategy (such as lowland rice) reduce their metabolism and preserve carbohydrates until the flood recedes, facilitating the regrowth after the submergence phase (Yeung et al., 2019). Contrastingly, plants adopting the LOES strategy (e.g., deepwater rice) enhance shoot elongation and/or bending of leaves (hyponasty) to reach the water surface and restore air contact or develop new plant structures such as aerenchyma or adventitious roots. However, both strategies involve a deep and complex metabolic rearrangement to help hypoxic tissues survive.

At the physiological level, one of the earliest effects of soil flooding is the reduction of root water uptake associated to a decrease in root hydraulic conductance (Else et al., 2009; Rodríguez-Gamir et al., 2011). Under these conditions, stomatal closure, presumably regulated by abscisic acid (ABA), is triggered to prevent dehydration (Arbona and Gómez-Cadenas, 2008; Hsu et al., 2011). To this respect, the identification of the HCR1 kinase has allowed the identification of the putative genetic link between plant responses to hypoxia mediated by the RAP2.12 transcription factor and the plant water status (Shahzad et al., 2016). Moreover, several members of the Raf-like MAPKKK family have been recently identified as activators of *SUCROSE NON-FERMENTING 1-RELATED SUBFAMILY 2* type kinases (SnRK2s) which have been described as key regulators of plant responses to water stress in an ABA-dependent as well as ABA-independent manner (Shinozawa et al., 2019). This establishes a promising link between sensing perturbations of water and oxygen balances induced by soil flooding and downstream ABA signaling (Soma et al., 2020; Takahashi et al., 2020; Figure 1C). Hence, high basal transpiration rate could be an important factor defining soil flooding tolerance and subsequent recovery (Arbona et al., 2009; Yeung et al., 2018). Arabidopsis *abi2-1* mutant with higher stomatal transpiration due to impaired ABA signaling showed enhanced plant survival after submergence (Bui et al., 2020). In addition, differential recovery after submergence in Arabidopsis was associated to the activity of three genes: *RESPIRATORY BURST OXIDASE HOMOLOG D (RBOHD)*, *SENESCENCE-ASSOCIATED GENE113*, and *ORESARA1*, which function in a regulatory network involving ROS burst upon de-submergence and the hormones ABA and ET. Indeed, recent evidence shows enhanced RBOHD expression in hypoxic tissues from ABA-deficient plants and increased biosynthesis of metabolites involved in anaplerotic pathways (Ala, Asp, Glu, and oxalacetate) potentially contributing to an improved ability for survival under oxygen-depleted conditions (De Ollas et al., 2021). This regulatory module controls ROS homeostasis, stomatal aperture, and chlorophyll degradation during submergence recovery (Yeung et al., 2018). Therefore, a response network involving ET, ABA, and downstream responses associated with modulation of metabolism, ROS generation, and aquaporin function in hypoxic and aerated tissues could be envisaged.

## Plant Hormonal Control Of Low-O_2_ And Recovery Responses

Plant morphological adaptations to soil waterlogging are largely regulated by ET signaling which inhibition or blockage results in total or partial abolition of these traits (Jackson, 2008; Hattori et al., 2009; Negi et al., 2010; Vidoz et al., 2010). However, as in any other plant developmental or adaptative process, ET does not exert its activity alone but interacting with other hormonal factors such as gibberellins (GAs), auxin (IAA), NO, or ABA (Voesenek and Bailey-Serres, 2015). To this respect, ABA has long been acknowledged as the main regulator of plant responses to abiotic constraints such as drought, salinity, high temperature stress, etc. Moreover, emerging roles for ABA as a regulator of complex plant responses such as the combination of abiotic-biotic stresses have been reported (Ximénez-Embún et al., 2018; Arbona et al., 2020). However, its exact role on plant responses to low oxygen conditions, including abiotic factors that induce hypoxic or anoxic conditions to plant roots, has been partially over shaded by ET and other plant regulators.

Early works on soil flooding responses back in 1991 focused on ABA being the main mediator of tolerance to anoxia through the induction of specific protein biosynthesis, particularly alcohol dehydrogenase, which was associated to the improved tolerance of flooded corn seedlings (Hwang and Vantoai, 1991). However, these metabolic responses, aimed at keeping energy production in the partial or total absence of oxygen as well as the development of specific adaptations have been later linked to ET signaling (Geisler-Lee et al., 2010; Hess et al., 2011). In other plant species, such as the flooding-tolerant *Rumex palustris*, rice, or soybean, morphological adaptations such as leaf and petiole elongation or secondary aerenchyma formation despite being induced by ET also involved ABA as a negative regulator (Benschop et al., 2005; Steffens and Sauter, 2005; Shimamura et al., 2014). In hypoxic tissues, ET induces the expression of ABA 8'-hydroxylases genes resulting in the active reduction of ABA levels (Pan et al., 1998; Saika et al., 2007; Chen et al., 2010; Hsu et al., 2011; Arbona et al., 2017) and the accumulation of its catabolite phaseic acid (PA; Arbona et al., 2017; Figure 1C). This reduction of ABA levels promotes underwater leaf growth in rice and *R. palustris* (Benschop et al., 2005; van Veen et al., 2013) and adventitious root formation in wheat, rice, and tomato associated to the repression of GA signaling and the induction of polar auxin transport (Steffens et al., 2006; Dawood et al., 2016; Nguyen et al., 2018). The regulation of the ABA catabolism in plant tissues subjected to hypoxia results controversial. In one hand, it was reported that ET or submergence induced the reduction of ABA levels at expenses of PA accumulation in *R. palustris* (Benschop et al., 2005), suggesting its ET-regulated active degradation. However, in Arabidopsis, ET did not induce any significant alteration in the expression of genes involved in ABA metabolism, as extracted from the analysis of several Arabidopsis ET receptor mutants (Bakshi et al., 2018). In this sense, recent evidence from the watercress *Nasturtium officinale*, showed that early ABA decline in flooded plants did not seem to be regulated by ET or GA (Müller et al., 2019). Taken together, this evidence could indicate that although ABA depletion *via* its targeted degradation is a common feature in hypoxic plant tissues, it might not be necessarily under ET control and could be independently regulated in a species-specific manner.

At the molecular level, the central module or *core* of the ABA signaling pathway is based on phosphorylation/dephosphorylation events, which include the ABA intracellular receptors PYRABACTIN RESISTANCE1 (PYR1)/PYR1-LIKE(PYL)/REGULATORY COMPONENTS OF ABA RECEPTORS (RCAR), the clade A of phosphatases type-2C (PP2Cs) and the ABA-dependent kinases SnRK2.2, SnRK2.3, and SnRK2.6/OST1 (Fujii et al., 2009; Cutler et al., 2010). At low ABA levels, clade A PP2Cs interact with SnRK2.2, SnRK2.3, and SnRK2.6/OST1 kinases resulting in their dephosphorylation that blocks their catalytic activity. However, the increase of ABA levels leads to the formation of the ABA-PYR/PYL/RCAR-PP2C ternary complex that inhibits the phosphatase activity of PP2Cs which releases SnRK2s that are activated by upstream kinases or autophosphorylation to phosphorylate different downstream target elements such as transcription factors and ion channels (Melcher et al., 2009; Figure 1C). Interestingly, analysis of the PYR/PYL/RCAR-PP2C ternary complex formation indicate that some functional specialization of ABA receptors to preferentially inhibit certain PP2Cs in the presence of ABA exists, suggesting their potential to integrate fluctuating hormone levels into the ABA-response pathway (Antoni et al., 2012; Tischer et al., 2017). Moreover, SnRK2s phosphorylation by upstream kinases has been shown as a crosstalk point to modulate ABA-signaling (Cai et al., 2014).

The concomitant upregulation of ABA receptors and genes that contain *cis*-acting ABRE and DRE elements in their promoter regions during hypoxic conditions has been described in soybean and, recently, in tomato (De Ollas et al., 2021), supporting the role of ABA in the regulation of responses of plants to low-O_2_ (Nakayama et al., 2017). In line with this, upregulation of the citrus PYR/PYL/RCAR ABA receptors *CsPYL4* and *CsPYL5* and the ABA-dependent genes *CsABI5* and *CsRD22* suggested an active ABA signaling response in hypoxic tissues despite the overreduction in the hormone levels (Arbona et al., 2017). Moreover, the Arabidopsis Raf-like MAPKKK *HRC1* which is involved in the low-O_2_ signaling pathway in roots may require the formation of the ABA-PYR/PYL/RCAR-PP2C ternary complex that blocks the PP2Cs activity at the same time that it produces the activation of the ABA-dependent SnRK2s by phosphorylation (Shahzad et al., 2016; Takahashi et al., 2020). In addition, Arabidopsis mutants impaired in ABSCISIC ACID INSENSITIVE4 (ABI4) transcription factor activity also showed hindered systemic responses under hypoxia, which indicated the importance of an intact ABA signaling pathway to sustain plant systemic responses (Hsu et al., 2011). Hyponastic growth under hypoxic conditions in Arabidopsis was affected by ABA as extracted from studies in *aba2-1* and *aba3-1* mutants or fluridone-treated wild type plants showing higher petiole angles upon ET treatment. ABA-hypersensitive mutants or ABA-treated wild type plants showed a significantly lower petiole angle, suggesting an antagonistic activity of ABA and ET in the induction of hyponastic growth (Benschop et al., 2007). Moreover, tomato plants impaired in ABA biosynthesis also showed no stomatal response upon imposition of soil waterlogging but, interestingly, ABA deficiency did not affect soil flooding-induced accumulation of *ACC SYNTHASE* and *ACC OXIDASE* gene transcripts, suggesting no effect on the ability for ET biosynthesis (De Ollas et al., 2021). In different plant experimental systems, upregulation of gene expression in hypoxic roots (including ABA-dependent genes) was proportional to the ability for ABA biosynthesis, in spite of hormone levels decline, supporting the positive role of this hormonal response (Arbona et al., 2017; De Ollas et al., 2021). Interestingly, the ABA insensitive *abi2-1* mutant which is refractory to the PYR/PYL/RCAR-mediated inhibition of the ABI2 phosphatase showed enhanced plant survival after submergence (Bui et al., 2020), which implicates ABA also in plant recovery after desubmergence. In barley grains, hypoxia (either imposed by incubating de-hulled seeds at 5% O_2_ or mimicked by the presence of glumellae) increased expression of HvABA8OH1 hydroxylase involved in ABA catabolism and genes participating in ABA signaling such as ABI4 or VIVIPAROUS1(VP1/ABI3), contributing to maintain dormancy (Mendiondo et al., 2010). Deficient mutants in PRT6 or ATE1 activities which result in the stabilization of N-degron substrates showed increased ABA sensitivity in germination assays (Mendiondo et al., 2016), probably as a result of increased PYR/PYL/RCAR ABA receptors expression that enhances the inhibition of the PP2Cs activities. Nevertheless, soil flooding induced accumulation of ABA-derived PA (Arbona et al., 2017) could constitute itself a signaling event as this molecule has been recently shown *in vitro* to interact with and activate, in the submicromolar range, a subset of the ABA PYR/PYL/RCAR receptor family members leading to an apparently selective activation of the ABA signaling pathway, subsequently adding more complexity and plasticity to the regulatory network (Weng et al., 2016). However, the physiological relevance of the PA as a signaling molecule in hypoxic tissues still needs to be evaluated.

**Figure 2:**
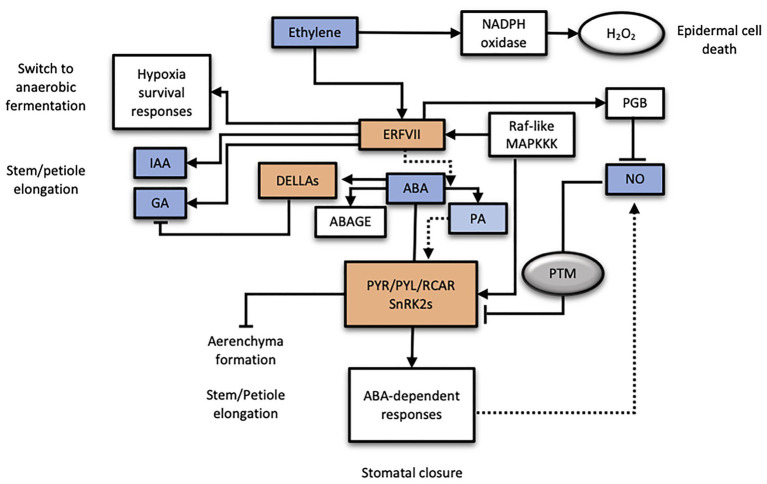
Working model of the ABA hormone under low oxygen conditions and its interaction with other plant hormones. Plant hormones involved are indicated in blue color, whereas molecular key players of different hormone signaling pathways are highlighted in orange color. PTM, protein post-translational modifications.

Different sources of evidence point toward the interaction of several hormonal signaling pathways participating in plant responses to hypoxia/anoxia such as ABA, ET, GAs, NO, or IAA (Voesenek and Bailey-Serres, 2015; Figure 2). In flooding-tolerant *R. palustris*, petiole elongation is related to apoplast acidification involving expansin and xyloglucan endo-transglycosylase/hydrolase (XTH) activation, pointing to IAA signaling. In addition, it also involves the activation of GA signaling through phytochrome interacting factors (PIF) and the repression of ABA synthesis and signaling (Voesenek and Bailey-Serres, 2015). On the contrary, in the flooding-intolerant *Rumex acetosa*, no differences in endogenous ABA and GA levels occur during submergence and also sensitivity of plants to GAs is significantly reduced. However, as extracted from *ACC OXIDASE* gene expression, ethylene production seemed to be active and maintained in both plant species (van Veen et al., 2013). Similarly, in rice seedlings, submergence induced the degradation of ABA which, indirectly, induced GA signaling (Voesenek and Bailey-Serres, 2015). In this network of interactions, brassinosteroids have also been involved as modulators downstream to SUB1A-mediated ethylene signaling (Sasidharan and Voesenek, 2015), potentially inducing SLENDER1 AND 2 (SLR1 and 2) DELLA proteins. Adventitious root formation in this plant species, requires promotion of epidermal cell death to allow protrusion of roots followed by their emergence and elongation, processes that are induced by ET and GAs and inhibited by ABA (Steffens et al., 2006, 2012). In soybean, soil flooding-induced formation of secondary aerenchyma was reduced by ABA (Shimamura et al., 2014) but, at the same time, promoters of the stress-upregulated genes were enriched in *cis*-acting ABA-responsive elements (Nakayama et al., 2017). This suggests that ABA activation signaling occurs despite the reduction in ABA levels and the impaired development of adaptations to soil flooding.

In recent years, NO has arisen as an important modulator of different plant processes such as germination and responses to abiotic stresses including low oxygen conditions (Prakash et al., 2018). In plants, NO levels are balanced by its biosynthesis, catalyzed by nitrate reductase, and scavenging, carried out by phytoglobins (Becana et al., 2020). Under hypoxic conditions, NO depletion is regulated by ET through RAP2.3 ERFVII transcription factor in Arabidopsis (León et al., 2020) which, in turn, facilitates the stabilization of ERFVII transcription factors pre-adapting plants to survive to hypoxia (Hartman et al., 2019). NO has been shown to act downstream ABA signaling in the regulation of seed dormancy, stomatal conductance (at least partially; Kolbert et al., 2019) and also in the responses to desiccation through its interaction with phytoglobins (Rubio et al., 2019). Moreover, it has been shown that NO might antagonize ABA signaling through post-translational Tyr nitration of PYR/PYL/RCAR ABA receptors that significantly reduced their PP2CA inhibitory activity in the presence of the hormone (Castillo et al., 2015) and Gibbs et al. (2014) reported that NO and O_2_ in Arabidopsis seeds promote the degradation of ERFVII, leading to the downregulation of ABI5 and germination. Conversely, recent evidence supported the involvement of ABA, at least partially, in the induction of phytoglobins and NO biosynthesis genes expression in hypoxic conditions (De Ollas et al., 2021), pointing toward a complex crosstalk interaction between these two factors and ET. To make this story even more complex, it has been recently reported that NO-dependent overexpression of several genes associated to ABA signaling in Arabidopsis requires an intact N-degron pathway (León et al., 2020), which supports the role of stabilized ERFVII as negative regulators of ABA-responsive genes. All these loads of evidence make necessary to examine ET, NO, and ABA crosstalk more specifically at the gene and tissue levels considering each experimental model used (seedlings vs. seeds).

## Working Model For ABA Signaling Under Low Oxygen and Reoxygenation Conditions

The current working model for ABA signaling under low oxygen conditions is summarized in Figure 2. It starts with the ABA level depletion in an apparent ET-dependent manner by the activation of 8'-ABA hydroxylase and ABA UDP-glycosyl transferase B2 activities. Both reduced ABA or increased PA levels could act as a positive signal efficiently channeled through specific PYR/PYL/RCAR ABA receptors with differential affinity for both ligands. A specific ABA-PYR/PYL/RCAR-PP2C complex formation, along with the induction in receptor expression, could account for a highly specific ABA signaling module under hypoxic conditions which may be reinforced by the putative O_2_ selective activation of the ABA-dependent SnRK2s mediated by the Raf-like HRC1 kinase. In turn, any effect on ABA signaling impacts on GA signaling as ABA promotes the stabilization of negative regulator DELLA proteins (Ross et al., 2011). In flooding-tolerant genotypes, complete submergence triggers adaptive responses that involve an intricate interaction of plant hormones, although the specific interaction seems to be particular of each plant species. For instance, in flooding tolerant *R. palustris*, ABA signaling is repressed by ET allowing GA-induced petiole elongation, whereas in the flooding-intolerant *R. acetosa*, this did not naturally happen. In deepwater rice, however, stem elongation is regulated by direct induction of GA biosynthesis carried out by ERFs SK1 and 2 (Hattori et al., 2011). Growth of adventitious roots at the nodes of rice stems in response to partial submergence involves ET and the activation of NADPH oxidases that induce H_2_O_2_ production in apparently independent pathways (Steffens and Sauter, 2009). Interestingly, internode elongation in rice induced by ET was also accompanied by a drastic reduction in ABA levels as a result of its degradation (Saika et al., 2007). However, in other plant models such as water cress (*Nasturtium officinale*), evidence suggests a direct involvement of ABA in underwater stem growth and discards the participation of ET or GAs (Müller et al., 2019). The available information allows stating that ABA depletion is a common feature in hypoxic plant tissues whereas the nature of the signal triggering this reduction still remains elusive as extracted from different plant systems (Arabidopsis, Citrus, or water cress). Moreover, data support the soil flooding-specificity of this ABA decline (Arbona et al., 2017) and its role as a positive signal (Arbona et al., 2017; De Ollas et al., 2021). Taken together, results suggest the uncoupling of ABA catabolism from ET signaling constituting a putative independent hypoxia signaling pathway, potentially inducing specific responses. Moreover, the modulation of ABA signaling by other pathways such as NO or ET, at least marginally, should be further investigated as potential modulators of tolerance to hypoxic conditions in non-adapted plant species.

## Author Contributions

MG-G and VA conceived and wrote first draft of the manuscript. MG-G, AG-C, and VA revised and edited subsequent versions of the manuscript. All authors contributed to the article and approved the submitted version.

### Conflict of Interest

The authors declare that the research was conducted in the absence of any commercial or financial relationships that could be construed as a potential conflict of interest.
